# Prenatal alcohol exposure and offspring mental health: A systematic review

**DOI:** 10.1016/j.drugalcdep.2019.01.007

**Published:** 2019-04-01

**Authors:** Kayleigh E. Easey, Maddy L. Dyer, Nicholas J. Timpson, Marcus R. Munafò

**Affiliations:** a UK Centre for Tobacco and Alcohol Studies, School of Psychological Science, University of Bristol, UK; b MRC Integrative Epidemiology Unit, University of Bristol, UK; c Population Health Sciences, Bristol Medical School, University of Bristol, UK

**Keywords:** Alcohol, Mental health, Pregnancy, Prenatal, Intrauterine, Systematic review

## Abstract

**Background:**

High levels of alcohol use in pregnancy have been shown to be associated with negative physical health consequences in offspring. However, the literature is less clear on the association of alcohol use in pregnancy and offspring mental health, specifically for low levels of prenatal alcohol exposure. We conducted a systematic review to evaluate studies examining this association.

**Methods:**

Studies were identified by searching PsycINFO, PubMed and Web of Science, and were included if they examined alcohol use during pregnancy as an exposure and offspring mental health at age 3 or older as an outcome. We excluded non-English language publications and studies of fetal alcohol syndrome.

**Results:**

Thirty-three studies were included and were categorized by mental health outcomes: anxiety/depression, emotional problems, total internalizing problems, total problem score, and conduct disorder. Over half of the analyses reported a positive association of prenatal alcohol exposure and offspring mental health problems.

**Conclusions:**

Our review suggests that maternal alcohol use during pregnancy is associated with offspring mental health problems, even at low to moderate levels of alcohol use. Future investigation using methods that allow stronger causal inference is needed to further investigate if these associations shown are causal.

## Highlights

• Prenatal alcohol use is associated with offspring mental health problems.
• There is disparity in the measurement of internalising disorders across studies.
• Future studies should utilise methods that allow stronger causal evidence.

## 1 Introduction

Maternal health behaviors during pregnancy, such as tobacco and alcohol use, are associated with adverse offspring health consequences. In particular, heavy alcohol use has been shown to cause physical and cognitive impairments (Bille et al., 2007; Sayal, 2007; Walthall et al., 2008), as well as, Fetal Alcohol Syndrome (FAS) (Mukherjee et al., 2006). Despite evidence of the harmful effects of alcohol use during pregnancy, it remains common, particularly at low levels (O’Keeffe et al., 2015). Whether such light to moderate alcohol use during pregnancy may affect offspring outcomes is less clear. A recent meta-analysis found that only a small number of prospective studies have investigated the association of light to moderate maternal alcohol use in pregnancy with offspring outcomes (Mamluk et al., 2017). This meta-analysis focused on pregnancy outcomes such as gestational diabetes and childhood outcomes related to FAS, such as behavioral problems and cognitive impairment. The authors describe the lack of evidence for either a harmful effect or for a safe level of intrauterine alcohol exposure and highlight the poor quantity and quality of contributing studies.

The effects of light to moderate alcohol use in pregnancy on non-physical, behavioral and mental health outcomes are even less clear. Some studies have reported that maternal alcohol use during pregnancy is associated with various negative outcomes, such as increased levels of conduct and depressive disorders in offspring (Disney et al., 2008; O’Connor, 2001). However, light alcohol use in pregnancy has also been reported to be associated with improved outcomes (i.e., appears protective). Kelly et al. (Kelly et al., 2012) found that drinking 1–2 units of alcohol in pregnancy was associated with higher cognitive abilities in male offspring at age five, with worse offspring outcomes observed for abstainers and heavy drinkers. As the authors found drinking to be socially patterned, with mothers who reported light alcohol use more likely to be from higher income households and with better education, these findings may, therefore, be due to residual confounding. Robinson et al. (Robinson et al., 2010) found no evidence that light alcohol use is a risk factor for offspring mental health problems up to age 14. However, the authors note that this finding may be due to sample attrition within the cohort.

There are also important methodological differences across studies, such as the way that mental health is measured. Some studies report only a total internalizing or externalizing disorder score, without showing how the subscales of each item (such as anxiety or depression) contribute individually (Robinson et al., 2010). Without a standard measure used across studies, differences in methods introduce substantial heterogeneity and mean that comparison or replication of findings becomes problematic. Of the research that is available, many studies report outcomes for young age groups, showing the impact prenatal alcohol exposure may have during the developmental stages of childhood only. However, it is less clear how prenatal alcohol exposure may affect offspring mental health as the child becomes older, and if any associations shown at earlier ages persist into adulthood.

We therefore conducted a systematic review of the existing literature, to determine the association of alcohol use in pregnancy with subsequent mental health in offspring aged three and above.

## 2 Method

### 2.1 Selection strategy

This review was conducted according to PRISMA (Preferred Reporting Items for Systematic Reviews and Meta-analyses) guidelines (Moher et al., 2009), and was preregistered on the Open Science Framework (osf.io/yrn2r). Electronic databases (PsycINFO, PubMed and Web of Science) were searched until mid-March 2017 to identify English language publications.

Screening of study eligibility was conducted by one reviewer (KEE) and irrelevant articles excluded based on title and abstract. Full-text articles were subsequently reviewed to determine eligibility, with reasons for exclusion documented for each paper. A 10% check of all the articles found at each of these stages were completed by a second reviewer (MLD), and any disagreements on eligibility were discussed and resolved by mutual consent.

### 2.2 Eligibility criteria

The search strategy included keywords related to “pregnancy”, “alcohol”, and “mental health” (see supplementary materials). At the initial stage of extraction, studies were excluded if they were review articles or animal studies. As the association between heavy drinking and FAS are well established, studies which only reported FAS outcomes were also not included. This was to further refine the review away from clinical diagnoses of FASD and potentially heavier alcohol exposures during pregnancy. Many of the FASD symptoms have a strong externalizing component also, and this review sought to focus on the effects on internalizing disorders. However, it is noted that FASD is underdiagnosed and therefore, the studies included may still be representing offspring with undiagnosed FASD, despite efforts to limit this.

Any source of mental health measure was included (e.g., self-report or maternal report). Outcomes measured below the age of three were excluded also, as we were interested in offspring outcomes at older ages.

### 2.3 Data extraction

Data were extracted by one reviewer (KEE) on study location, design, maternal age during pregnancy, offspring gender, and age at outcome measures were used to assess alcohol use in pregnancy and mental health outcomes in offspring, as well as any covariates used within the study. A 100% check on the data extraction was conducted by a second reviewer (MLD).

If studies reported multiple alcohol exposures from varying stages of pregnancy, the earliest time point was extracted. Where multiple alcohol exposure types (e.g., cumulative or binge drinking) were used, the cumulative alcohol amount was extracted. If studies reported mental health outcomes at multiple ages, results from the oldest age group were extracted. Fully adjusted results are presented when reported in studies. If included studies reported multiple mental health outcomes, the data were extracted separately for each outcome to allow for investigation of which individual subscale of mental health is most strongly associated with intrauterine alcohol exposure. Data from sensitivity analyses, such as splitting analyses by sex were not extracted.

### 2.4 Rationale for not conducting meta-analysis

Within the pre-registered protocol, a meta-analysis was planned if deemed appropriate from the included studies. However, a meta-analysis was not conducted as there were substantial differences between studies in exposure measurement, time to follow up, location, covariates used, and frequency of outcomes sampled. As a meta-analysis was not possible, we have instead presented an appraisal of the current literature, enabling the reader to be aware of the limitations in interpretation, and further provided suggestion for how future studies may improve the synthesis of evidence.

Eligible studies were included if they contained the desired outcome and exposure variables within their data set, which meant the included studies were not always initially designed to investigate associations between prenatal alcohol exposure and offspring mental health.

## 3 Results

### 3.1 Characteristics of included studies

**Fig. 1:**
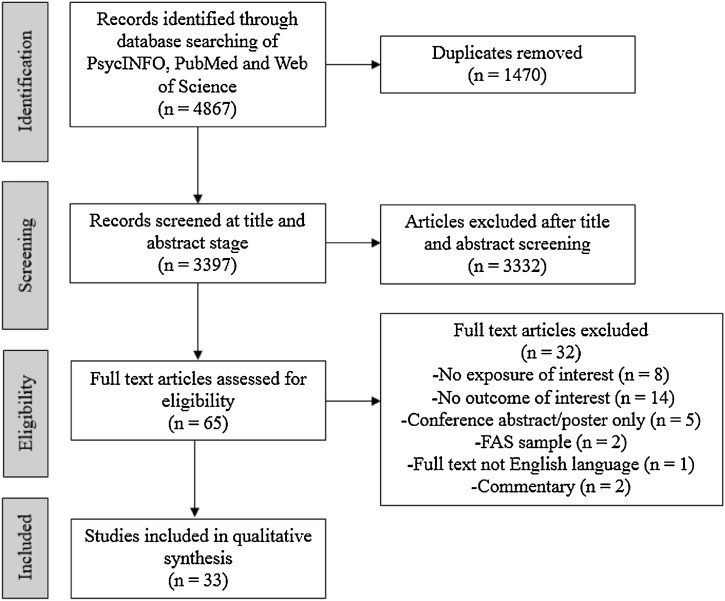
Flowchart of search strategy.

The initial search identified 3397 articles (after removal of duplicates), of which 65 were chosen for full text review after exclusion of irrelevant studies based on title, abstract and keywords. Of these, 32 did not meet inclusion criteria and were excluded (see Fig. 1). Thirty-three articles met the inclusion criteria, details of which are shown in Table 1. Six studies used a UK population, 17 American, 5 Australian, 3 Scandinavian, 1 Canadian, and 1 Taiwanese. Details of excluded studies are shown in Supplementary Table 1.

**Table 1:** Included studies.

Author/Year	Country	Substance use measure (continuous/categorical)	Gestation period measured	Mental health measure (name)	Mental health type (anxiety/depression)	Method of mental health measure (self-report/clinical assessment)	Summary of results presented in the paper
(Alvik et al., 2013)	Norway	Categorical: Binge drinking during weeks 0-6 (never; <once a week; ≥ once a week)	0-6 weeks	SDQ	Total problem score, emotional, conduct	Parent report	*Total problems: OR (CI), p* <once a week 1.5 (1.0 to 2.1), 0.05 ≥ once a week 4.1 (1.7 to 9.8), <0.01 *Emotional*: ≥ once a week 3.2 (1.3 to 8.0), <0.05 *Conduct:* ≥ once a week 3.0 (1.3 to 7.2), <0.05
(Bada et al., 2007)	USA	Categorical: yes/no	Doesn't say	CBCL	Internalising, total problem score	Researcher administered	*Internalising: Mean difference (CI), p* 0.61 (-0.01 to 1.24), 0.06 *Total scores:* 0.78 (0.08 to 1.47), .03
(Brown et al., 1991)	USA	Categorical: never drank, stopped drinking, continued to drink after education intervention	Doesn't say	CBCL	Internalising, anxious, depressed, total problem score	Teacher & maternal report	*Internalising: F, p* 2.04, ns *Anxious* *<1, ns* *Depressed* *4.25, 0.02* *Total* 7.16, 0.002
(Chiu et al., 2009)	Taiwan	Categorical. At least once a week, not	Whole pregnancy	CBCL	Anxiety/depression	Parental report	*ns*
(D’Onofrio et al., 2007)	USA	Continuous: Mean number of days exposed to alcohol per week in pregnancy	Whole pregnancy	CBCL	Conduct problems	Mother report	b = 0.06, p < 0.05, SE 0.02
(Day et al., 2013)	USA	Continuous: Average daily volume of alcohol	First trimester	Adult Self-report (continuation of the CBCL)	Internalising, total problem score	Self-report	*Internalising: coefficient, r^2^, p* 1.65, 0.004, <0.05 *Total problem score:* 1.9, 0.01, <0.05
(Disney et al., 2008)	USA	Categorical: drank in pregnancy, or not	Whole pregnancy	Structured Clinical Interview for DSM-III-R personality disorders	Conduct disorder	Mother and child report	*Coefficient, p.* 11.59, <.001
(Fryer et al., 2007)	USA	Categorical. Yes or no to alcohol use in pregnancy. Historical records	Whole pregnancy	Kiddie Schedule for Affective Disorders and Schizophrenia for school aged children, present and lifetime version and the Computerised diagnostic interview schedule for children, version IV.	Depressive disorders, conduct disorder, generalised anxiety disorder	Parent & child report	*Point estimate (CI), p* Depressive disorder: 0.18 (0.08 to 0.31), <.05 Conduct disorder: 0.15 (0.01 to 0.28), p<.05 GAD: 0.08 (0 to 0.18), p > 0.05
(Graham et al., 2013)	USA	Categorical: exposed (>4drinks per occasion at least once), or not	Whole pregnancy	CBCL	Internalising	Parent report	*F* (1266) = 17.83, p<.001
(Hill et al., 2000)	USA	Categorical: median split of consumption	1^st^, 2^nd^ and 3^rd^ trimester	KSADS- Schedule for Affective Disorders and Schizophrenia for school aged children.	Depression, anxiety, conduct disorder, total problem score	Parent, child and psychiatrist report	*OR (CI), p* Depression: 4.48 (1.45 to 13.83), 0.009 Anxiety: 3.27 (1.13 to 9.38), .028 Conduct: 4.42 (1.35 to 14.33), 0.014
(Kelly et al., 2009)	UK	Categorical: Never, light (not more than 1-2 units pw/per occasion), moderate (not more than 3-6units pw, 3-5 units per occasion), heavy/binge (≥7 units pw or >6 units per occasion)	Asked retrospectively after birth (child 9 months)	SDQ	Conduct, emotional, total problem score	Parent report	*Boys: OR (CI)* *Total*: Light 0.77 (0.56 to 1.07) Moderate 0.65 (0.35 to 1.23) Binge 1.76 (0.83 to 3.73) *CD*: Light 0.59 (0.44 to 0.81) Moderate 0.68 (0.39 to 1.21) Binge 0.53 (0.22 to 1.27) *Emotional*: Light 0.85 (0.60 to 1.21), Moderate 0.81 (0.40 to 1.64) Binge 2.15 (1.09 to 4.25) Girls: *Total*: Light 0.70 (0.43 to 1.14) Moderate 1.18 (0.63 to 2.19) Binge 0.83 (0.30 to 2.28) *CD*: Light 0.72 (0.52 to 1.00) Moderate 1.60 (0.92 to 2.78) Binge 1.18 (0.49–2.83) *Emotional*: Light 0.95 (0.65 to 1.38) Moderate 0.90 (0.45 to 1.79) Binge 1.62 (0.72 to 3.68)
(Kendler et al., 2013)	USA	Doesn’t state	18 and 32 weeks gest	SDQ	Conduct disorder & emotional problems	Maternal report	*Beta, SE, p* *Conduct:* *0.052, 0.014, <0.001* *Emotional: 0.038, 0.014, <0.01*
(Knopik et al., 2009)	USA	Categorical: 1-10 days, 11-35 days, >35 days, some heavy use (at least 5-6 drinks on days and at least once a month), frequent heavy use (5+ drinks on 2-3 days pm)	Whole pregnancy	DICA for telephone administration	Conduct disorder	Self-report	*Beta, SE, p* *1-10 days use: -.001, .024* *11-35 days use: -.077, .058* *>35 days use: .095, .132* *Some heavy use: -.053, .093* *Frequent heavy use: .388, .158, p<.01*
(Larkby et al., 2011)	USA	Categorical: none (adv = 0), light (adv≤0.4), moderate (>0.4, ≤0.89), heavy (>0.89)	1 st trimester	DIS-IV	Conduct disorder	Structured interview	*First trimester:* p = 0.002
(Murray et al., 2016)	UK	Categorical: Moderate drinking (>0-6 units pw at any time in pregnancy), and not drinking >6 units pw on a single occasion at any time in pregnancy	18 and 32 weeks gest	SDQ	Conduct disorder	Self-report	*p* = 0.633
(Niclasen et al., 2014b)	Denmark	Cumulative intake of alcohol	Whole pregnancy	SDQ	Internalising	Parent report	Boys OR (CI) 0: 1.03 (0.99 to 1.08) >0-5: 1.02 (0.98 to 1.06) 1.02 (0.99 to 1.06) >15-45: ref >45-90: 0.99 (0.96 to 1.03) >90: 0.92 (0.88 to 0.97)
(Niclasen et al., 2014a)	Denmark	Categorical: binge drinking (5+) drinks in early, or late pregnancy, and never	16 and 30 weeks gestation	SDQ	Conduct, emotional, internalising	Parent report	*Internalising: relative change in mean (CI)* Early binge 1.00 (0.98 to 1.03) Late binge 1.05(0.89 to 1.24) *Conduct: OR (CI)* Early binge 1.01 (0.91 to 1.11) Late binge 0.81 (0.49 to 1.43) *Emotional: OR (CI)* Early binge 0.93 (0.84 to 1.03) Late binge 0.86 (0.51 to 1.57)
(O’Connor and Kasari, 2000)	USA	Categorical: Abstinent-light (0-2 drinks per occasion), moderate-heavy (3 or more drinks per occasion)	Whole pregnancy	Pictorial Depression Scale	Depression	Self-report	*t (39) = 2.02, p < 0.05*
(O’Connor and Paley, 2006)	USA	Continuous: Maximum drinks per drinking occasion	Whole pregnancy	Pictorial Depression Scale	Depression	Self-report	*r* = 0.35, *p*<0.05
(O’Connor, 2001)	USA	Continuous: Maximum drinks per occasion	Whole pregnancy	Pictorial Depression Scale	Depression	Self-report	*r* = .43, *p*<.01
(O’Leary and Bower, 2009)	Australia	Categorical: abstinent, low (over a week <7 drinks AND on any day no more than 1-2 standard drinks), moderate (10 g of alcohol per occasion) daily, heavy (5 or more per occasion)	1^st^ trimester	CBCL	Anxiety/depression, internalising, total problem score	Parent report	*OR (CI)* *Anxiety/depression*: Low 1.06 (0.59 to 1.88) Moderate 2.24 (1.16 to 4.34) Heavy 2.82 (1.07 to 7.43) *Internalising:* Low 1.04 (0.73 to1.49) Moderate 1.14 (0.67 to 1.94) Heavy 2.65 (1.36 to 5.14) *Total:* Low 0.97 (0.69 to 1.37) Moderate 1.17 (0.74 to 1.84) Heavy 1.62 (0.85 to 3.11)
(O’Leary et al., 2010)	Australia	Categorical: abstinent, <10 g alcohol per occasion, 10 g alcohol daily & binge drinking < weekly, binge (60-70 g per week).	1^st^ trimester	CBCL	Anxiety/depression	Parent report	*First trimester OR (CI)* Low 1.06 (0.59 to 1.88) Moderate 2.24 (1.16 to 4.34) Heavy 2.82 (1.07 to 7.43)
(Robinson et al., 2010)	Australia	Categorical: abstinent, occasional (up to 1 drink pw), light (2-6 drinks pw), moderate (7-10 drinks pw), heavy (≥11 drinks pw)	18 weeks gestation	CBCL	Total problem score, internalising	Parent report	*OR (CI), p* *Internalising* *18 weeks* Occasional: 0.85 (0.67 to 1.07) 0.164 Light: 0.57 (0.42 to 0.76), <0.001 Moderate: 0.31 (0.14 to 0.69), 0.004 Heavy: 0.76 (0.33 to 1.76), 0.519 *Total* Occasional: 0.82 (0.63 to 1.06), 0.133 Light: 0.63 (0.46 to 0.86), 0.003 Moderate: 0.43 (0.21 to 0.88), 0.020 Heavy: 0.68 (0.31 to 1.47), 0.323
(Sayal et al., 2007)	UK	Categorical: <1 glass pw, ≥1 glass pw	18 weeks gestation	SDQ	Conduct disorder	Parent and teacher report	*OR (CI)* *82 months* <1 glass: 1.18 (0.99 to 1.40) ≤1 glass: 1.20 (0.95 to 1.52)
(Sayal et al., 2009)	UK	Categorical: ≥ 4 drinks in a day on any one occasion, < 4 drinks in a day on any occasion	18 and 32 weeks gestation	SDQ	Conduct problems, total problem score	Parent report	*Conduct. Coefficient (CI), p* *0.12 (0.02 to 0.22), 0.020* *Total* *0.36 (0.04 to 0.68), 0.026*
(Sayal et al., 2013)	UK	Categorical: <1 glass pw, ≥1 glass pw	18 weeks gest	SDQ	Conduct disorder, total problem score	Parent report	*Conduct. Coefficient (CI), p* <1 glass: 0.06 (-0.02 to 0.14), 0.151 ≥1 glass: 0.04 (-0.07 to 0.15), 0.462 *Total* <1 glass: 0.13 (-0.14 to 0.40), 0.347 ≥1 glass: 0.04 (-0.33 to 0.42), 0.825
(Sayal et al., 2014)	UK	Categorical: Binge drinking. Consumed <4 drinks (includes non-drinkers), and ≥ 4drinks, at any time in pregnancy	18 and 32 weeks gestation	SDQ	Conduct disorder, total problem score	Parent and teacher report.	*Conduct. Coefficient (CI), p* 0.05 (-0.06 to 0.15), 0.406 *Total* 0.30 (-0.07 to 0.67), 0.109
(Silva et al., 2015)	Australia	Categorical: yes/no	Whole pregnancy	Doesn't say	Depression, anxiety	Parent report	*OR (CI)* Depression: 1.08 (0.55 to 2.12) Anxiety: 1.14 (0.66 to 1.97)
(Sood et al., 2001)	USA	Categorical: Average absolute alcohol pd across pregnancy. Low (>0-<0.3 fl oz/pd), moderate/heavy (≥0.3 fl oz)	Whole pregnancy	CBCL	Internalising and total score	Parent report	*R^2^, β, p* Internalising: 0.274, 0.096, 0.020 Total score: 0.308, 0.114, .005
(Staroselsky et al., 2009)	Canada	Documented history of alcohol exposure in utero, or not	Not stated	CBCL	Anxiety and depression	Not stated	*ns*
(Tearne et al., 2015)	Australia	Categorical: ≤10 drinks pw, 11> drinks pw	18 weeks gest	CBCL	Total problem score, internalising	Parent report	*ns*
(Walthall et al., 2008)	USA	Categorical: Exposure levels of <1 standard drink in gestation, drank in pregnancy	Whole pregnancy	National Institute for Mental Health Computerized Diagnostic Interview Schedule for Children-Fourth Edition	Disorders of: Separation anxiety, generalised anxiety, Conduct Disorder, major depressive disorder	Parent or caregiver report	*F, p, β, B(SE), r^2^(adjusted r^2^)* *Separation anxiety disorder*: 9.82, <.001, 0.24, 13.35(5.62), 0.13(.12) *Generalised anxiety disorder*: 6.27, 0.001, 0.17, 8.35(4.18), 0.09(.08) *CD:* 9.69, <.001, 0.24, 7.62(3.19), 0.24(.21) *Major depressive disorder:* 6.27, <.001, 0.15, 6.98(4.89), 0.17(.14)
(Ware et al., 2013)	USA	Categorical: Control: no more than 1 drink pw on average, and never more than 2 drinks on any single occasion, >4 drinks at least once in pregnancy	Whole pregnancy	Computerised Diagnostic Interview Schedule for Children-IV and CBCL	Anxiety/depression, withdrawn/depression, Internalising, total problem score	Parent completed	*Anxious depressed:* F = 9.70, p = .002. *Withdrawn/depressed:* F = 9.25, p = .003. *Internalising*: F = 22.32, p < 0.001. *Total problems:* F = 46.61, p < 0.001.

PAE: prenatal alcohol exposure, pm: per month, pd: per day, *ns*: reported as “non-significant”.

### 3.2 Summary of results

Studies ranged in sample size from 41 to 37,315, and length of follow up from 3 to 26 years. Of the 33 included studies, 23 (70%) reported using male and female participants, 1 (3%) reported only using females and 9 (27%) did not report the sex of the participants.

The associations described refer to a positive association (e.g., intrauterine alcohol exposure was associated with increased depression) unless stated otherwise.

### 3.3 Assessment tools used

The exposure of prenatal alcohol use was measured using a binary or categorical measure for 30 of the 33 included studies. Of these, 4 used a binary exposure to measure alcohol consumption during pregnancy (yes/no). The remaining 26 studies all used varying categorical exposures, with different definitions of “low”, “moderate” and “binge” alcohol exposure used between studies (see Table 1). The 3 studies that did use a continuous measure of drinking, all measured different types of alcohol exposure (e.g., average daily volume of alcohol, cumulative alcohol intake across pregnancy, maximum number of drinks per occasion).

Ten studies used the Strengths and Difficulties Questionnaire (SDQ) (Goodman, 1997, 2001) as the primary measure of offspring mental health, 13 studies used the Child Behavioral Checklist (CBCL) (Achenbach, 1991), 3 used the Pictorial Depression Scale (O’Connor and Kasari, 2000), 1 used the Structured Clinical Interview for DSM-III R Personality disorders (Spitzer et al., 1987), 1 used the Diagnostic Interview for Children and Adolescents (DICA) for telephone administration (Reich, 2000), 1 used the Diagnostic Interview Schedule for DSM-IV (Robins et al., 2000), 1 used the Kiddie Schedule for Affective Disorders (KSADS) (Chambers et al., 1985), 1 used the National Institute for Mental Health Computerized Diagnostic Interview Schedule for Children Version IV (NIMH DISC-IV) (Shaffer et al., 2000), 1 used both the KSADS and NIMH DISC-IV combined, and 1 study did not report the measure used.

Due to the different types of scales/measures used across studies, we categorized studies on the type of mental health outcome they reported measuring: Anxiety/depression (measures of anxiety, depression, withdrawn/depressed, generalized anxiety disorder, separation anxiety and major depression were combined due to the limited number of studies using each individual scale and their comorbidity), emotional problems, total internalizing score, total problem score, and conduct disorder. The percentages of associations reported below are indicative of the total number of studies included within each outcome subscale.

The 33 studies included in this review included ten varying measures of assessing mental health, seven of which were used within only one study each. To aid interpretation of the literature, we sought to create a categorization system that captured every subscale used by the studies in our review. This was guided by the Strengths and Difficulties Questionnaire and Child Behavior Checklist, which was used for outcome measurement for the majority of studies (23/33; 67%). This was not an effort to generate a new categorization system, but to clarify the coverage of existing literature. Only select studies reported the “total scores” of either internalizing or total problem scores and are reported in this review when available in each paper. Such total problem scores are derived from the individual mental health subscales also presented. However, description of both the total problem scores and individual subscales are given within this review to allow a more comprehensive overview of the findings reported.

#### 3.3.1 Anxiety/depression

A total of 13 studies investigated the association of maternal prenatal alcohol exposure with subsequent offspring anxiety/depression. Of these studies, 9 (69%) found evidence to support a positive association of increased maternal prenatal alcohol exposure and increased offspring anxiety/depression (*n* = 41 to 1327), and 4 (31%) found no evidence of an association (*n* = 11 to 321). Of the 9 studies reporting a positive association, 6 of these studies investigated a population with either low socioeconomic status (SES) or offspring with other presenting mental health problems such as attention deficit hyperactive disorder (ADHD). Of the 4 studies reporting no clear evidence of an association, 3 utilized a sample of offspring with a diagnosed mental health problem, or from a family with a history of having an alcohol problem. The remaining study that did not find an association had a small sample of only 11 mothers who consumed alcohol during pregnancy and may have been underpowered to detect an association.

#### 3.3.2 Emotional problems

A total of 4 studies investigated the association of maternal prenatal alcohol exposure with subsequent offspring emotional problems. Of these studies, 2 (50%) found evidence to support a positive association (*n* = 1003, 228), and 2 (50%) found no clear evidence of an association (*n* = 9460, 29,529). All 4 studies that reported an outcome of emotional problems were longitudinal population-based cohorts. Two were Scandinavian (one in Norway found a positive association, one in Denmark found no clear evidence of association), one UK-based (no clear association), and one US-based (positive association).

#### 3.3.3 Total internalizing problems

A total of 11 studies investigated the association of maternal prenatal alcohol exposure with subsequent offspring total internalizing problem scores. Of these studies, 5 (45%) found evidence to support a positive association (*n* = 272 to 607), and 1 (9%) found evidence to support a negative association (*n* = 2370). The remaining 5 studies (45%) found no clear evidence of an association (*n* = 54 to 371,525). Of the 5 studies reporting a positive association, 4 studies used a sample with either low SES, offspring with an ADHD diagnosis, or a family history of having an alcohol problem. The one study that reported a negative association used a sample from a Western Australian pregnancy cohort, in which social disadvantage predicted loss to follow up (14 years later). This study, therefore, represented a sample with higher SES. Of the 5 studies reporting no association, one of these also used participants from the Western Australian cohort. One used a sample of pregnant women with low SES who were offered interventions to reduce alcohol consumption during pregnancy. This study had a low sample size of 54 women and may have been underpowered. One sampled children who were prenatally exposed to cocaine. The remaining two studies used participants from the Danish National Birth Cohort.

#### 3.3.4 Total problems

A total of 15 studies investigated the association of maternal prenatal alcohol exposure with subsequent offspring total problem scores. Of these studies, 8 (53%) found evidence to support a positive association (*n* = 54 to 8240), and 1 (7%) found evidence to support a negative association (n = 2370). The remaining 6 studies (40%) found no clear evidence of an association (*n* = 150 to 3460). Of the 8 studies that reported a positive association, 2 used a sample with low SES, 1 recruited participants based on having ADHD and high alcohol exposure, and one recruited a sample with cocaine exposure, and one study oversampled on mothers with high alcohol consumption. The remaining 3 studies were longitudinal studies of samples from high income countries with sample sizes ranging from 1003 to 8240. The one study that found a negative association used participants from a Western Australian pregnancy cohort and were a higher SES sample. Of the 6 studies that did not report an association, 1 also used the Western Australian pregnancy cohort, 4 used UK based longitudinal cohorts, and the remaining study recruited participants at high or low risk of an alcohol problem based on familial history. The one study that reported negative associations between light drinking and offspring total internalizing problems and total problem scores, also reported no evidence of an association between heavy drinking and offspring total internalizing problems. The sample size of heavy drinking (11 or more drinks per week) within this study (Robinson et al., 2010) was small (*n* = 42), and may, therefore, have been underpowered to detect a true association.

#### 3.3.5 Conduct disorder

A total of 17 studies investigated the association of maternal prenatal alcohol exposure with subsequent offspring conduct disorder. Of these studies, 9 (53%) found evidence to support a positive association (*n* = 69 to 8621), and 1 (6%) found evidence to support a negative association (*n* = 9460). The remaining 7 studies (41%) found no evidence of an association (*n* = 150 to 29,529). Of the studies that reported a positive association, 2 used a sample of children with either social skills deficits or ADHD and heavy alcohol exposure, and 1 used a cohort of children being treated at a psychiatric facility. The remaining 6 studies were population-based studies from Western countries, with sample sizes ranging from 69 to 8621. The one study that reported a negative association used a UK based cohort study, with a large sample size of 9460. Of the 7 studies that reported no association, 5 of these studies used the same UK based cohort, the Avon Longitudinal Study of Parents and Children (ALSPAC). Of the remaining two studies, one used a sample of the Danish birth-cohort and one recruited participants that were either low or high risk of having an alcohol problem, defined through familial history of alcohol problems.

Two studies used participants from the same cohort (Robinson et al., 2010; Tearne et al., 2015) yet reported contrasting directions of associations with the same measured outcomes (total problems scores; total internalizing scores). This may be due to different samples from the same cohort being analyzed; both studies controlled for varying covariates resulting in different sample sizes. Each study also measured the original continuous alcohol exposure using separate methods. One study created a binary alcohol exposure measure of ≤ 10 drinks per week compared to > 10 drinks per week (Tearne et al., 2015), and the other created a categorical measure consisting of 5 categories of weekly alcohol consumption (Robinson et al., 2010).

Of the studies that measured total problem scores as the outcome, four studies from the same first author reported using samples from the ALSPAC cohort yet only one study reported a positive association (Sayal et al., 2009), with the remaining three reporting no clear association. This may be due to different exposure measures being used between the studies. One study (Sayal et al., 2009) created a binary measure of binge drinking (≥ 4 units a day) and is, therefore, measuring drinking patterns and not drinking frequency as 2 other studies were (Sayal et al., 2013, 2007). The remaining study using the same cohort (Sayal et al., 2014) also measured binge drinking; however, they investigated the association with an older age group (11 years) compared to the 2009 study (7 years).

## 4 Discussion

The aim of this systematic review was to investigate the association between maternal alcohol use during pregnancy and offspring mental health, by appraising the current literature and describing the findings. In general, our findings suggest that alcohol use during pregnancy is associated with increased risk of mental health problems in the offspring, specifically anxiety/depression, total problems and conduct disorder. Of the five extracted outcome types, three types of mental health (anxiety/depression, total problems and conduct disorder) showed a majority reporting a positive association. An equal number of studies reported both a positive association and no clear evidence to support an association, between maternal alcohol use in pregnancy and emotional problems, as well as total internalizing scores. Only two studies showed that increased alcohol exposure during pregnancy was associated with *increased* positive mental health in offspring. In one of these studies (Kelly et al., 2009), the authors suggest that the J-shaped curve shown in their results may not actually be due to light drinking in pregnancy causing a reduction in offspring mental health, but instead due to residual confounding.

There are limitations that should be considered when interpreting these results. First, as all the included studies are observational, their findings may still be influenced by the well described problems of residual confounding. Measures of potential confounders differed greatly across studies, meaning it was not possible to assess any consistent effects from varied confounding. Of note is the different approach to adjustment for maternal drug use during pregnancy across studies. Bada et al. (Bada et al., 2007) assessed children prenatally exposed to cocaine and adjusted for alternative illicit drug use such as opiates and marijuana; however, few other studies included prenatal drug use and those that did mainly adjusted for marijuana use only. Second, varying methods were used for exposure and outcome measurements between studies. Of the 33 studies included in this review, all but four used a different measure of prenatal alcohol use, with varying definitions of “low” or “moderate” alcohol exposure. As there is no universally accepted definition of low, moderate or heavy alcohol use in pregnancy (Sood et al., 2001), this makes comparisons between studies difficult. This substantial heterogeneity between studies meant that a meta-analysis was inappropriate for this review. Differences were also shown across studies for the method of report for prenatal alcohol exposure (e.g., self-report, medical report), and at what timepoint alcohol use was recorded (e.g., early or late pregnancy, after birth). Therefore, it cannot be concluded from this review at which stage of pregnancy maternal alcohol use has the greatest effect on offspring mental health. Third, there was substantial variation in the length of follow up times (3 to 26 years). The study that measured the oldest age group within this review (Day et al., 2013), found intrauterine alcohol exposure was associated with total problem scores in offspring at a mean age of 22, which suggested the associations shown at earlier ages may be permanent. However, replication using older age groups is required to confirm this, as all other studies within this review except for one (Larkby et al., 2011) investigated a sample of offspring aged 16 or younger. Fourth, sample sizes ranged from 41 to 37,315 offspring, and some of the smaller studies may have been underpowered. Both the amount and type of confounders that were adjusted for also varied greatly between studies, making comparisons across studies difficult when assessing confounding influences. Different diagnostic tests with varying cut-offs for determining clinical thresholds were used to assess offspring mental health, measured by self-report, parental/carer report or teacher report. Although some studies within this review used the same measures, they did not always report every subscale within each test. For example, the CBCL measures a variety of subscales, but often studies only utilized the total summed score. This made it further difficult to assess which subscale of internalizing disorders may be contributing to the total score, which is why the individual subscales are presented individually as well as any reported total score.

The current review describes and summarizes the findings for published literature investigating maternal prenatal alcohol exposure and offspring mental health. It also details the limitations in being able to create a synthesis of results due to the marked differences in exposure, and outcome measurement across studies, including types of measures/subscales used, method of report and length of follow up. We propose that future studies within this area should aim to use a detailed measure of alcohol frequency across trimesters, instead of simply a binary measure of the presence/absence of alcohol use at any point during gestation. This would allow the reader to infer the amount of alcohol and timing of exposure which may be associated with offspring outcomes. This may also enable a synthesis of results in a meaningful meta-analysis. The inclusion of similar outcome measurements to previous research would also be advantageous, however, due to the limitations in available measurements within studies, it is instead suggested that future studies describe the findings for each subscale within internalizing measures, as opposed to merely stating ‘total’ scores. The current review also highlights the disparity in which age internalizing outcomes have been measured, with many focusing on younger age groups. Within studies this may be due to the younger age of available participants, however, with the length of follow up for many cohort studies now increasing, it is suggested that future studies also focus on older age groups to investigate if any associations shown at for earlier ages continue into adulthood and replicate those that have suggested it may (Day et al., 2013).

Only English language studies were included in this review, which may have led to the omission of some studies. However, it has been reported that little evidence of bias is introduced from the exclusion of non-English studies (Morrison et al., 2012). Studies were also only included if they were published. By not including unpublished studies this means that low quality studies were unlikely to have been included, however, this could mean that publication bias may have affected our results. If non-published studies were included, there may have been more null results.

Two of the outcome categories included an externalizing component (conduct disorder and total problem scores). Total problem scores were often calculated from the individual mental health subscales, and which subscales were included in this total varied across measures and studies. This means it is difficult to summarize how much of the total problem score is attributed purely to internalizing or externalizing disorders.

The longitudinal studies which were included within this review can identify associations but do not provide evidence of causality on their own. Future studies should, therefore, utilize methods that allow stronger causal inference, such as negative control analyses and Mendelian Randomization (MR) where possible. However, as genetic variants currently identified for alcohol use suffer from weak instrument bias and, therefore, have reduced power to detect a true effect, MR is not often a suitable approach in investigating the effect of prenatal alcohol exposure on offspring mental health. Negative control analyses can instead be used to show if an association is still observed by a different exposure that is likely to have a similar confounding structure to the original exposure of interest, but no biological link (Gage et al., 2016). If an association is also found within the negative control analyses, this is likely to be due to confounding and not due to the original exposure of interest (Davey Smith, 2008). When investigating the potential causal influence of maternal alcohol use in pregnancy on offspring outcomes, paternal alcohol use during pregnancy can be used as a negative control, as paternal alcohol use can have no direct biological effect on the developing fetus. Triangulation of multiple approaches (Lawlor et al., 2016) would allow researchers to investigate the causal effects of maternal alcohol use during pregnancy.

In summary, this review helps to address a gap in the literature by systematically reviewing and describing published research on intrauterine alcohol exposure and offspring mental health for all ages above 3. We found evidence of a positive association between maternal prenatal alcohol use and offspring mental health problems, specifically anxiety and depression, conduct disorder and total problem scores. As the alcohol exposures between studies were all measured using different scales, it is difficult to discern what level of intrauterine alcohol exposure is related to each mental health outcome. As this review excluded studies that measured FAS outcomes specifically, the novel design means we are more certain that the results obtained are for lower levels of alcohol use. However, as this review sought to evaluate the subclinical effects of alcohol use by excluding predefined groups with FAS, the current review still cannot be certain that the included studies are not still capturing offspring with undiagnosed FAS. This is due to a lack of formal categorization of how much intrauterine alcohol exposure is required to cause FAS and be clinically dangerous to the developing fetus. The exact relationship between FASD and ADHD remains unclear; however, ADHD is the most commonly reported mental health diagnosis for children exposed to maternal alcohol use during pregnancy (Fryer et al., 2007). Some studies included within this review recruited a sample of offspring with an ADHD diagnosis. As ADHD has been suggested to be a clinical subtype of FASD (Peadon and Elliott, 2010), this may mean that the inclusion of samples with ADHD diagnosis may actually have been capturing offspring with FASD.

Despite the high amount of heterogeneity across studies, and differences in study design we still evidenced a predictable positive association between low levels of alcohol exposure and offspring mental health problems. Such findings give support for future work to further investigate children with low levels of intrauterine alcohol exposure, as well as the need to focus on causal inference.

## Role of funding support

NJT is a Wellcome Trust Investigator (202802/Z/16/Z) and works within the University of Bristol NIHR Biomedical Research Centre (BRC) and CRUK Integrative Cancer Epidemiology Programme (C18281/A19169). MRM is a program lead in the MRC Integrative Epidemiology Unit (MC_UU_00011/7). KEE, MLD, NJT and MRM work in the Medical Research Council Integrative Epidemiology Unit at the University of Bristol which is supported by the Medical Research Council and the University of Bristol (this funds KEE’s PhD studentship).

## Contributors

We confirm that this manuscript contains original work that has not been previously published and is not submitted for publication elsewhere. All authors have agreed to the content and submission and have no conflicts of interest.

## Conflict of interest

No conflict declared.
