# Supplementary material for: Prenatal alcohol exposure and offspring mental health: A systematic review
Source: Drug Alcohol Depend. 2019 Apr 1;197:344–53. doi: 10.1016/j.drugalcdep.2019.01.007 (PMC6446223; doi:10.1016/j.drugalcdep.2019.01.007)
Supplement: Supplementary file 1 [file mmc1.docx]

**Supplementary Material for the Article:**

Prenatal alcohol exposure and offspring mental health:

A systematic review

**This material supplements, but does not replace, the peer-reviewed paper in**

***Drug and Alcohol Dependence*.**

Kayleigh E Easey^1,2^, Maddy L Dyer^1,2^, Nicholas J Timpson^2,3^, Marcus R Munafò^1,2^

1. UK Centre for Tobacco and Alcohol Studies, School of Psychological Science, University of Bristol, UK.
2. MRC Integrative Epidemiology Unit, University of Bristol, UK.
3. Population Health Sciences, Bristol Medical School, University of Bristol, UK

**Correspondence:**

Kayleigh E. Easey

Tobacco and Alcohol Research Group, School of Psychological Science

MRC Integrative Epidemiology Unit, University of Bristol, UK.

Email: kayleigh.easey@bristol.ac.uk

**Supplementary Table 1.** Studies excluded at full text stage.

| **Author** | **Year** | **Reason for exclusion** |
| --- | --- | --- |
| Alvik et al | 2011 | Exposures not relevant |
| Baglot et al | 2016 | Conference abstract/poster only |
| Barbier et al | 2008 | Conference abstract/poster only |
| Bhatara et al | 2006 | Exposures not relevant |
| Chasnoff et al | 2015 | Exposures not relevant |
| Chen | 2012 | Outcomes not relevant |
| Coles et al | 1997 | FAS sample |
| Delaney-Black et al | 1998 | Outcomes not relevant |
| Delaney-Black et al | 2000 | Outcomes not relevant |
| Enoch et al | 2016 | Outcomes not relevant |
| Hanna et al | 1997 | Outcomes not relevant |
| Howell et al | 2006 | Outcomes not relevant |
| Infante et al | 2015 | Outcomes not relevant |
| Skarpness et al | 2012 | Outcomes not relevant |
| Knopik et al | 2005 | Conference abstract/poster only |
| Kukla et al | 2008 | Not English language publication |
| Mick et al | 2002 | Outcomes not relevant |
| Motz et al | 2013 | Commentary |
| O’Connor et al | 2002 | Outcomes not relevant |
| O’Connor et al | 2002 | Exposure not relevant |
| Piper et al | 2014 | Exposure not relevant |
| Rasmussen et al | 2011 | Exposure not relevant |
| Rettew | 2008 | Commentary |
| Rodriguez et al | 2009 | Outcomes not relevant |
| Salom et al | 2014 | Exposures not relevant |
| Sato et al | 2008 | Conference abstract/poster |
| Sayal et al | 2007 | Commentary |
| Sciberras et al | 2011 | Outcomes not relevant |
| Smith | 2016 | Commentary |
| Sood et al | 2002 | Conference abstract/poster |
| Way et al | 2012 | FAS group |
| Willford et al | 2006 | Outcome not relevant |

FAS: Foetal alcohol syndrome

**Search terms used for review**

“mental health” OR depress* OR anxiety OR mood OR conduct OR internali?ing

AND

alcohol OR ethanol OR drink*

AND

pregnan* OR perinatal OR prenatal OR intrauterine OR utero OR f?etal OR gestation
